# Prevalence and factors associated with incomplete immunization of children (12–23 months) in Kwabre East District, Ashanti Region, Ghana

**DOI:** 10.1186/s13690-018-0315-z

**Published:** 2018-11-19

**Authors:** Anthony Wemakor, Gideon Kofi Helegbe, Alhassan Abdul-Mumin, Shadrack Amedoe, Jessica Adjoa Zoku, Ahimah Ivy Dufie

**Affiliations:** 1 Department of Nutritional Sciences, School of Allied Health Sciences, University for Development Studies, Tamale, Ghana; 2 Department of Biochemistry and Molecular Medicine, School of Medicine and Health Sciences, University for Development Studies, Tamale, Ghana; 3 Department of Paediatrics and Child Health, Tamale Teaching Hospital/School of Medicine and Health Sciences, University for Development Studies, Tamale, Ghana

**Keywords:** Immunisation coverage, Determinants of immunisation, Children, Ashanti Region, Ghana

## Abstract

**Background:**

Childhood immunization is one of the most cost effective health interventions but its rate has been declining recently in Ghana. Information on immunization coverage and determinants is needed to improve immunization programmes. The objective of this study was to determine the prevalence and factors associated with incomplete immunization of children (12–23 months) in Kwabre East District, Ghana.

**Methods:**

A cross-sectional, community-based survey involving 322 children and their mothers was carried out. Data were collected on socio-demographic characteristics of mothers, childhood immunization history and mothers’ knowledge and practices of immunization using a structured questionnaire. Children were classified as incompletely immunized if they failed to receive at least one of 8 vaccine doses: - one dose of Bacillus Calmette–Guérin (BCG), 3 doses each of pentavalent, 3 doses of polio and one dose of measles per WHO/UNICEF definition. Chi-square and logistic regression analyses were used to identify the factors associated with incomplete immunisation.

**Results:**

The prevalence of incomplete immunization was low (15.5%) suggesting high immunisation coverage but the coverage of the second measles dose, taken at 18 months of age, was the lowest (23.9%). Most of the mothers knew the importance of immunisation (95.7%) and at least one vaccine-preventable disease or symptom (84.9%). Two factors associated with incomplete immunisation in bivariate analyses (community of residence, and mother’s knowledge of number of oral polio vaccines given to children) were no longer significant in a logistic regression model. Compared to children in Aboaso, children in Gyamfi Wonoo (AOR = 1.81, 95% CI = 0.80–4.08), Mamponteng (Bonwunu) (AOR = 0.59, 95% CI = 0.24–1.48) and Mamponteng (Town) (AOR = 0.63, 95% CI = 0.26–1.55) had similar odds of incomplete immunisation. Similarly, mother’s lack of knowledge of the number of doses of polio vaccine given to children had no effect on the odds of incomplete immunisation (AOR = 0.53, 95% CI = 0.22–1.26).

**Conclusions:**

Immunization coverage is high in the Kwabre East district but very few children received the second measles dose. None of the maternal and child factors assessed is associated with immunisation coverage. Further research is needed to identify the determinants of immunisation coverage and the reasons for the low uptake of second measles dose in the study area.

**Electronic supplementary material:**

The online version of this article (10.1186/s13690-018-0315-z) contains supplementary material, which is available to authorized users.

## Background

Childhood immunization is one of the most cost-effective interventions in health care delivery [1]. In Ghana, the immunization schedule normally requires children to receive 16 vaccine doses from birth up to the age of 23 months [2] but in 2016, a new vaccination was added for protection against meningitis. Children therefore receive a dose of Bacille Calmette-Guerin (BCG) at birth; 4 doses of Oral Polio Vaccine (OPV) at birth, and at 6, 10 and 14 weeks of age; 3 doses of Diphtheria, Pertusis, Tetanus, Haemophilus influenzae type B and Hepatitis B (DPT/HiB/HepB) pentavalent vaccine at 6, 10 and 14 weeks of age, 3 doses of pneumococcal vaccine at 6, 10 and 14 weeks of age, and 2 doses of rotavirus vaccine at 6 and 10 weeks of age. Thereafter, they receive a dose each of yellow fever and combined measles/rubella vaccines at 9 months of age and a dose each of measles-only and meningitis A vaccines at 18 months of age.

Immunisation coverage is useful for monitoring the effectiveness of immunisation programmes [3]. In Ghana, the coverage of childhood immunisation had reduced nationally (and also by region) from 79 to 77% with Western Region recording the most reduction from 82 to 69% from 2008 to 2014 [2]. Within the same period the percentage of children that received none of the childhood immunisations increased from 1 to 2% nationally. In the Ashanti region, immunisation coverage reduced from 85% in 2008 to 79% in 2014 [2]. Monitoring immunisation coverage and determinants at sub-national levels is essential for prioritizing and tailoring immunisation campaigns and operational plans to address immunization gaps and reach every child with life-saving vaccines. The objective of this study was to determine the prevalence and factors associated with incomplete immunization of children (12–23 months) in Kwabre East District, Ashanti Region, Ghana.

## Methods

A community-based cross-sectional study involving children aged 12–23 months and their mothers/caregivers was conducted in four communities of Mamponteng (Bonwunu), Mamponteng (Town), Gyamfi Wonoo, and Aboaso in four sub-districts in Kwabre East District, Ashanti Region, Ghana. The study period covered January and February, 2017.

### Sample size determination and sampling

The minimum required sample size was determined using the single population formula by Snedecor and Cochran n = (t)^2^*(p)(q)/(d)^2^, where n = sample size, t = reliability coefficient associated with a selected confidence level, p = estimated proportion of an event of interest, q = p-1, and d = desired margin of error. Using the population size of children aged 11–23 months in the four study communities (*n* = 792), the coverage of complete immunization in children 11–23 months in the Ashanti Region of 79.0% [2], the reliability coefficient associated with 95% confidence level of 1.96, and the margin of error of 5% a minimum sample size of 192 was estimated. This was multiplied by 1.5 to account for cluster sampling and increased by 10% to obtain the final sample size of 322. The four communities were randomly sampled from four sub-districts and the number of children sampled from each sub-district was proportional to the total number of children 11–23 months in that sub-district. The number of children sampled in the four communities were 65, 89, 79 and 89 for Gyamfi Wonoo, Mamponteng (Town), Aboaso, and Mamponteng (Bonwunu) respectively.

Mother-child pairs were selected using systematic random sampling. The sampling interval for each community was obtained by dividing the total number of children in that community by the number of sample required from that community. The starting point was selected randomly from the sampling interval and the sampling interval was added until the required number of children was obtained from the community.

Data were collected using a structured questionnaire on socio-demographic factors of mothers and children, immunization history of children and mother’s knowledge and practices of immunization. An immunisation card served as the main source of immunisation history of the child. The questionnaire consisted of both close and open-ended questions prepared based on literature review of previous work done on the subject. The questionnaire written in English was translated into Akan and back-translated into English independently by two persons very proficient in both the English and Akan languages to ensure the accuracy of the translation. Misunderstandings and unclear wordings identified in each case were corrected. The questionnaire was then pre-tested with 50 mother-child pairs in another community with similar characteristics as the study communities and fine-tuned further where necessary. The questionnaire was administered in Akan, the local language spoken in the study area, by three final year students of the Department of Nutritional Sciences, University for Development Studies, Tamale, who speak both English and Akan fluently. These students were trained in questionnaire administration, had excellent understanding of the questionnaire, and participated in the pre-testing of the questionnaire. A supervisor sampled and interviewed some of the interviewees and had comparably the same findings as the enumerators during the pre-testing.

### Study variables

#### Incomplete immunisation

According to the World Health Organisation/UNICEF Guidelines, complete immunization is defined as receipt of one dose of BCG, 3 doses of pentavalent, 3 doses of polio and one dose of measles within the first year of life [4]. Based on this definition any child who did not receive all these 8 vaccine doses before 12 months of age was classified as incompletely immunized. This is the dependent variable of the study.

#### Household socio-economic status

To assess socio-economic status of households, information was collected on the availability in the households of 14 items: mobile phone, TV set, satellite dish, sewing machine, mattress, radio, DVD player, bicycle, motorcycle, refrigerator, personal computer, electric fan, animal drawn cart, and car. Each household item was scored “1” if present in the household otherwise “0”. Using principal component analysis, a wealth score was derived for each household and the scores were grouped and divided into tertiles: low, medium and high tertiles (See Additional file 1).

#### Maternal knowledge on immunisation score

To summarize mothers’ knowledge on immunisation, the responses to 6 of the immunisation knowledge questions (i.e., importance of immunisation) were coded and scored. For each of the questions, a correct response was awarded a score of “1” and an incorrect response a score of “0” and all the scores were summed up to give the summary score. The summary score was then divided at midpoint into 2 categories i.e., “low” (0–3 scores) and “high” (4–6 scores).

### Data analysis

Data were analyzed using Stata (version 13). The association between maternal and child factors and child immunization status was explored using Chi-square test and logistic regression analysis. Factors that were statistically significant in bivariate analyses with Chi-square were entered into a logistic regression model in order to identify the independent determinants of incomplete immunization. In all analyses, a *p*-value less than 0.05 was considered statistically significant.

### Ethical considerations

Ethics permission for the study was granted by the Ethics Committee of the School of Medicine and Health Sciences/School of Allied Health Sciences, University for Development Studies, Tamale, Ghana. Informed consent was obtained from study participants before interviews were conducted. It was made clear to the study participants that they had the right to withdraw their participation at any time during data collection and that the information being collected will be kept confidential, accessed only by members of the research team and used only for the purposes of this research.

## Results

### Socio-demographic characteristics of mothers and children

A total of 322 mothers and their children were surveyed. The ages of the mothers ranged from 17 to 55 years with a mean of 29.9 (95% confidence interval = 29.3–30.5) years and most (34.2%) were in the age range 30–34 years (Table 1). The study subjects consisted predominantly of Christians (82.6%) and married mothers (83.5%) and most (35.7%) were of low household wealth tertile. About halve of the children were males (51.2%) and in the age range 12–15 months (45.0%).

**Table 1:** Socio-demographic factors of parents and children

Socio-demo graphic characteristic	Frequency (N)	Percent
Age group of mother (years)		
< 25	55	17.1
25–29	102	31.7
30–34	110	34.2
> 34	55	17.1
Total	322	100.0
Religion		
Christian	266	82.6
Non-Christian	56	17.4
Total	322	100.0
Marital status		
Single	44	13.7
Married	269	83.5
Widowed	9	2.8
Total	322	100.0
Ethnicity		
Akan	182	56.5
Others	140	43.5
Total	322	100.0
Occupation		
Trading	179	55.6
Office work	10	3.1
Health work	6	1.9
Teaching	22	6.8
Housewife	52	16.1
Others	53	16.5
Total	322	100.0
Mother’s educational level		
No education	26	8.1
Primary	46	14.3
Middle/ JHS	150	46.6
SHS/ Vocational	66	20.5
Tertiary	34	10.6
Total	322	100.0
Number of household members		
1–4	188	58.4
> 4	134	41.6
Total	322	100.0
Household wealth index (tertile)		
Low	115	35.7
Medium	109	33.9
High	98	30.4
Total	322	100.0
No. of children less than 5 years in the household		
1	197	61.2
2	117	36.3
3+	8	2.5
Total	322	100.0
Community of residence		
Gyamfi Wonoo	65	20.2
Mamponteng (Town)	89	27.6
Aboaso	79	24.5
Mamponteng (Bonwunu)	89	27.6
Total	322	100.0
Age group of child (months)		
12–15	145	45.0
16–19	115	35.7
20–23	62	19.3
Total	322	100.0
Sex of child		
Male	165	51.2
Female	157	48.8
Total	322	100.0

### Immunisation coverage

Children are required to receive seven vaccine-specific immunisations in six rounds from birth up to 23 months. Immunisation rates for four vaccine-specific immunizations (polio, pentavalent, rotavirus and pneumonia) were at least 90.0% (range 90.1–96.9%) but the rate was about a fifth (23.9%) for measles-only vaccine taken at 18 months of age.

The coverage of immunisation increased from 94.7% at first round (at birth) to about 97.0% in rounds 2 (at 6 weeks), 3 (at 10 weeks) and 4 (at 14 weeks) then decreased to 93.5% in round 5 (at 9 months). The coverage of individual vaccine doses was highest (97.8%) for polio 1, pentavalent 1, pneumococcal 1 and rotavirus 1 followed by polio 3, pentavalent 3 and pneumococcal 3 (96.9%). The measles only vaccine taken at round 6 (18 months) had the lowest prevalence 23.9% (Table 2). Of all the children, 272 (84.5%) were fully immunized (i.e. received one dose of BCG, 3 doses each of pentavalent and polio and one dose of measles within first year of life), 50 (15.5%) were partially immunized, and none of the children failed to receive a single vaccine.

**Table 2:** Coverage of individual vaccine doses

Immunisation	Coverage (*N* = 322)
	N (%)
At Birth (Round 1)	
BCG	303 (94.1)
Polio 0	295 (91.6)
All	305 (94.7)
At 6 Weeks (Round 2)	
Polio 1	315 (97.8)
Pentavalent 1	315 (97.8)
Pneumococcal 1	315 (97.8)
Rotavirus 1	315 (97.8)
All	315 (97.8)
At 10 weeks (Round 3)	
Polio 2	314 (97.5)
Pentavalent 2	314 (97.5)
Pneumococcal 2	314 (97.5)
Rotavirus 2	314 (97.5)
All	314 (97.5)
At 14 Weeks (Round 4)	
Polio 3	312 (96.9)
Pentavalent 3	312 (96.9)
Pneumococcal3	312 (96.9)
All	312 (96.9)
At 9 Months (Round 5)	
Measles/rubella	298 (92.5)
Yellow fever	294 (91.3)
All	309 (93.5)
At 18 Months (Round 6)	
Measles	77 (23.9)

### Mothers’ knowledge and practices of immunization

A greater majority of the mothers (more than 90%) knew the importance of childhood immunisations (i.e., vaccines protect, build immunity, prevent infections, ensures good health), and symptoms of vaccine preventable diseases with 86.3% able to mention at least one vaccine preventable disease (Table 3). A little over two-thirds (70%) of the mothers knew when children were supposed to complete their immunisations but knowledge on the age at which children receive measles/rubella immunisation (33.2%) and the number of doses of oral polio vaccine (9.9%) was low. Using a summary score for immunisation knowledge, a high percentage of the mothers had knowledge on childhood immunization (66.5%). Majority of respondents indicated that health workers never used unpleasant words on them (66.5%), always communicated the next date of immunisation to them (72.0%), and most said they always explained the significance of childhood immunization (41.3%). On the practice of immunisation, 61.8% and 17.1% of mothers said their children ever experienced fever and abscess at injection site respectively.

**Table 3:** Mothers’ knowledge and practices of immunization

Immunization knowledge and practices	Frequency (N)	Percent (%)
Mother knew the purpose of immunisation		
Yes	308	95.7
No	14	4.3
Total	322	100.0
Mother mentioned at least one vaccine preventable disease		
Yes	278	86.3
No	44	13.7
Total	322	100.0
Mother mentioned at least one symptom of vaccine preventable illnesses		
Yes	378	86.3
No	44	13.7
Total	322	100.0
Mother knew age at which measles/rubella immunisation is given		
Yes	107	33.2
No	215	66.6
Total	322	100.0
Mother knew the number of doses of oral polio vaccine given to children		
Yes	32	9.9
No	290	90.1
Total	322	100.0
Mother knew age at which a child is supposed to be fully immunized		
Yes	235	73.0
No	86	27.0
Total	322	100.0
Knowledge of immunization score		
Low (0–3)	108	33.5
High (4–6)	214	66.5
Total	322	100.0
Child has ever developed fever following immunization		
Yes	199	61.8
No	123	38.2
Total	322	100.0
Child has ever developed abscess following immunization		
Yes	55	17.1
No	267	82.9
Total	322	100.0
Health worker used unpleasant words on mothers during immunization		
Always	14	4.3
Sometimes	87	27.0
Rarely	7	2.2
Never	214	66.5
Total	322	100.0
Health worker explained the significance of childhood immunisation to mothers		
Always	133	41.3
Sometimes	95	29.5
Rarely	8	2.5
Never	86	26.7
Total	322	100.0
Health worker clearly communicated the next date of immunisation to mothers		
Always	232	72.0
Sometimes	27	8.4
Rarely	4	1.2
Never	59	18.3
Total	322	100.0

### Factors associated with incomplete immunization

Bivariate analyses using Chi-square were performed to identify child-, mother- or household-related factors associated with immunization status of children. In these analyses the community of residence (χ^2^_(3)_ = 10.624, *p* = 0.014) and mother’s knowledge of number of oral polio vaccine doses (χ^2^_(1)_ = 4.298, *p* = 0.038) were found to be associated with immunization status (Tables 4 and 5), whiles mother’s marital status showed borderline significance (χ^2^_(1)_ = 3.9181, *p* = 0.048). No associations were obtained for child characteristics (sex and age), or other maternal and household characteristics evaluated.

**Table 4:** Association of socio-demographic factors with immunization status of children

Socio-demographic factor	Incomplete immunization			Test statistics
	No (%)	Yes (%)	Total	
Age group of mother (years)				
< 25	44 (80.0)	11 (20.0)	55	χ^2^ = 6.9540, df = 3, *P* = 0.073
25–29	82 (80.4)	20 (19.6)	102	
30–34	101 (91.8)	9 (8.2)	110	
> 34	45 (81.8)	10 (18.8)	55	
Religion of mother				
Christian	225 (84.6)	41 (15.4)	266	χ^2^ = 0.015, df = 1, *P* = 0.902
Non-Christian	47 (83.9)	9 (16.1)	56	
Marital status				
Currently married	232 (86.3)	37 (13.7)	269	χ^2^ = 3.9181, df = 1, P = 0.048
Not currently married	40 (75.5)	13 (24.5)	53	
Ethnicity				
Akan	155 (85.2)	27 (14.8)	182	χ^2^ = 0.1532, df = 1, *P* = 0.696
Others	117 (83.6)	23 (16.4)	140	
Occupation				
Trading	150 (83.8)	29 (16.2)	179	χ^2^ = 0.1392, df = 1, *P* = 0.709
Others	122 (85.3)	21 (14.7)	143	
Mothers’ educational level				
None	19 (73.1)	7 (26.9)	26	χ^2^ = 3.959, df = 4, *P* = 0.412
Primary	37 (80.4)	9 (19.6)	46	
Middle/JHS	129 (86.0)	21 (14.0)	150	
SHS/vocational school	57 (86.4)	9 (13.6)	66	
Household wealth index (tertile)				
Low	91 (79.1)	24 (20.9)	115	χ^2^ = 3.9055, df = 2, *P* = 0.142
Medium	95 (87.2)	14 (12.8)	109	
High	86 (87.8)	12 (12.2)	98	
Community				
Mamponteng (Bonwunu)	80 (89.9)	9 (10.1)	89	χ^2^ = 10.6245, df = 3, *P* = 0.014
Gyamfi Wonoo	47 (72.3)	18 (27.7)	65	
Mamponteng (Town)	79 (88.8)	10 (11.2)	89	
Aboaso	66 (83.5)	13 (16.5)	79	
Number of household members				
> 4	109 (81.3)	25 (18.7)	134	χ^2^ = 1.7129, df = 1, *P* = 0.191
< = 4	163 (86.7)	25 (13.3)	188	
Number of children under five in household				
1	169 (85.8)	28 (14.2)	197	χ^2^ = 0.6688, df = 1, *P* = 0.413
2+	103 (82.4)	22 (17.6)	125	
Age group of child (months)				
12–15	126 (86.9)	19 (13.1)	145	χ^2^ = 1.4286, df = 2, *P* = 0.490
16–19	96 (83.5)	19 (16.5)	115	
20–23	50 (80.6)	12 (19.4)	62	
Sex of child				
Male	134 (81.2)	31 (18.8)	165	χ^2^ = 2.74, df = 1, *P* = 0.098
Female	138 (87.9)	19 (12.1)	157	

*Df* Degrees of freedom

**Table 5:** Association of maternal knowledge and practices of immunization with immunization status of children

Immunization knowledge and Practices	Incomplete Immunisation			Test statistics
	No (%)	Yes (%)	Total	
Mother mentioned at least one vaccine preventable disease				
Yes	236 (84.9)	42 (15.1)	278	χ^2^ = 0.2736, df = 1, *P* = 0.601
No	36 (81.8)	8 (18.2)	44	
Mother mentioned at least one symptom of vaccine preventable illnesses				
Yes	237 (85.3)	41 (14.7)	278	χ^2^ = 0.943, df = 1, *P* = 0.331
No	35 (79.6)	9 (20.4)	44	
Mother knew age at which measles/rubella immunisation is given				
Yes	95 (88.8)	12 (11.2)	107	χ^2^ = 2.2726, df = 1, *P* = 0.132
No	117 (82.3)	38 (17.7)	215	
Mother knew number of doses of oral polio vaccine given to children				
Yes	23 (71.9)	9 (28.1)	32	χ^2^ = 4.2985, df = 1, *P* = 0.038
No	249 (85.9)	41 (14.1)	290	
Mother knew age at which a child is supposed to be fully immunized				
Yes	204 (86.6)	31 (13.2)	235	χ^2^ = 3.619, df = 1, *P* = 0.057
No	68 (78.2)	19 (21.8	87	
Knowledge of immunization score				
Low (0–3)	88 (81.5)	20 (18.5)	108	χ^2^ = 1.1080, df = 1, *P* = 0.293
High (4–6)	184 (86.0)	30 (14.0)	214	
Mother have fears that vaccines can harm children				
Yes	47 (83.9)	9 (16.1)	56	χ^2^ = 0.0153, df = 1, P = 0.902
No	225 (84.6)	41 (15.4)	266	
Child has ever developed fever after immunisation				
Yes	173 (86.9)	26 (13.1)	199	χ^2^ = 2.4086, df = 1, *P* = 0.121
No	99 (80.5)	24 (19.5)	123	
Child has ever developed abscess after immunization				
Yes	45 (81.8)	10 (18.2)	55	χ^2^ = 0.3562, df = 1, *P* = 0.551
No	227 (85.0)	40 (15.0)	267	
Health worker used unpleasant words on mothers during immunization				
Always/sometimes	83 (82.2)	18 (17.8)	101	χ^2^ = 0.5903, df = 1, *P* = 0.442
Rarely/never	189 (85.5)	32 (14.5)	221	
Health worker explained the significance of childhood immunisations to mothers				
Always/sometimes	197 (86.4)	31 (13.6)	228	χ^2^ = 2.221, df = 1, *P* = 0.136
Rarely/never	75 (79.8)	19 (20.2)	94	
Health worker clearly communicated the next date for immunisation to mothers				
Always/sometimes	222 (85.7)	37 (14.3)	259	χ^2^ = 1.557, df = 1, *P* = 0.212
Rarely/never	50 (79.4)	13 (20.6)	63	

*Df* Degrees of freedom

In multivariate analysis, factors significant in bivariate analyses with incomplete immunization i.e., community of residence and knowledge of oral polio vaccine doses did not remain significant (Table 6).

**Table 6:** Multivariate analysis of factors associated with incomplete immunization in bivariate analyses

Variable	Adjusted Odds Ratio	95% Confidence Interval	*P*-value
Community of residence			
Aboaso	1.00		
Gyamfi Wonoo	1.81	0.80–4.08	0.156
Mamponteng (Bonwunu)	0.59	0.24–1.48	0.266
Mamponteng (Town)	0.63	0.26–1.55	0.317
Mothers knew the number of doses of oral polio vaccine given to children			
Yes	1.00		
No	0.53	0.22–1.26	0.148

## Discussion

We estimated the prevalence rate of incomplete immunization of children (12–23 months) and sought to identify its maternal and child-related determinants in Kwabre East, Ghana. Almost a sixth (15.5%) of the children surveyed were incompletely immunized suggesting high immunization coverage but among antigen-specific immunisations, measles had the least prevalence (23.9%).

The percentage of children fully immunized in this study population (84.5%) is better than both the Ashanti Regional and national coverage rates of 78.9% and 77.0% respectively [2] but lower than observed for children in Techiman (89.5%) [5]. Our rate is slightly higher than the 80.0% coverage rate recommended for districts by 2020 by the Global Vaccine Action Plan [6]. On the African continent, our rate is comparable to a coverage rate reported for children in Nigeria (84.9%) [7] but not for children in Kenya (76.6%) [8]. Apart from measles, which is taken at ages 9 and 18 months, the coverage of the individual vaccines was equally very high. Immunisation coverage is an important indicator of population health and a measure of the quality of health services provided [9]. The 2014 Ghana Demographic and Health Survey has indicated declining immunization rates [2] and this situation may have alerted individual districts (including study district) to increase their efforts in order to increase coverage. The low prevalence of measles-specific immunisation rate may be attributable to the long period (i.e., 9 months) between the first and second measles doses at rounds five and six respectively. In one study done in India the coverage of measles decreased from 34.9% (at first dose) to 22.7% (at second dose) [10].

We assessed mothers’ knowledge and practices of immunization. Generally, the mothers’ immunisation-related knowledge is high and most mothers know the importance of immunization for children, vaccine-preventable diseases and their symptoms, and the age at which children are to complete immunisation. This is against the backdrop that about two-thirds of the women had education up to only Junior High School level and it is known that formal education tends to positively influence health knowledge and behaviour [11]. The percentage of mothers who mentioned correctly the age at which children should complete immunization (73.0%) was higher than reported for mothers in Nigeria (14.1%) [12] and in India (52.4%) [13]. Again, our study recorded a higher percentage of mothers (95.7%) who knew the benefits of immunisation compared to a study done in India (82.0%) [14]. Overall, knowledge on immunization is high and the proportion of mothers (66.5%) with this knowledge can be compared to the 70.0% of mothers reported to be knowledgeable on immunization in Nigeria [15]. The mothers also have positive attitude towards the health workers reporting that they did not use unpleasant words on them. The observations in this study area suggest a combination of maternal interest in child health, availability of child preventive health services and friendly health workers in the District.

The study evaluated a range of maternal and child socio-demographic characteristics and maternal immunisation-related knowledge and practices as they have been shown to predict immunisation coverage [5, 16–19]. Among the factors evaluated, the community of residence and mothers’ knowledge of the number of doses of polio vaccines were significant only in bivariate analysis. Although we found a higher prevalence of incompletely immunized children belonging to unmarried mothers compared to married mothers (24.5% versus 13.7%), this did not reach statistical significance (*p* = 0.048) in bivariate analysis. Overall, we failed to find a link between maternal/child socio-demographic characteristics, household factors or immunization-related knowledge of mothers and children’s immunization status. This may reflect a lack of association between these factors and child immunisation status in the study area or the lack of sufficient power by the study to identify child immunisation determinants.

Our study has some limitations that should be considered in interpreting its findings. The use of a cross-sectional study design in which both the immunisation coverage and determinants were assessed at the same time meant that a cause-effect relationship cannot be established. We employed cluster sampling but did not take that into consideration in the analysis. Consequently, we computed 95% confidence intervals for means instead of standard deviations. Our sample size may have been inadequate to identify the determinants of immunisation, if there were, for our study population. Given the high immunisation coverage in the District, children who were not completely vaccinated may have been underrepresented and this may have underpowered the study. Despite these limitations we believe our study provides some insights into the coverage and determinants of childhood immunisation in the Kwabre East District and the findings may be relevant and generalizable to communities in the District with similar characteristics as the study communities.

## Conclusions

In exception of the second dose of measles vaccine taken at 18 months of age, the coverage of childhood immunisation in the Kwabre East District is high. However, our study did not find any association between maternal and child socio-demographic characteristics, household factors or immunisation-related knowledge of mothers and coverage of childhood immunisation. Mothers generally have high level of knowledge on childhood immunization. Further studies are required to identify the determinants of incomplete immunisation and to understand the low uptake of the second measles dose in the study area. Education campaigns should focus on the importance of the measles vaccine to improve the uptake of its second dose.

## Additional file

Additional file 1: Principal component analysis of household items to create a household wealth index. This is a Stata output on the use of principal component analysis to create a household wealth index. (TXT 6 kb)

## Abbreviations

BCG: Bacillus Calmette–Guérin
OPV: Oral Polio Vaccine
